# Supplementary material for: Iterative improvement in the automatic modular design of robot swarms
Source: PeerJ Comput Sci. 2020 Dec 7;6:e322. doi: 10.7717/peerj-cs.322 (PMC7924708; doi:10.7717/peerj-cs.322)
Supplement: Supplemental Information 3 [file peerj-cs-06-322-s003.zip › argos3/doc/api/standalone/a00464.html]

ARGoS: plugins/robots/foot-bot/simulator/footbot\_entity.cpp File Reference


- Main Page
- Related Pages
- Namespaces
- Classes
- Files

- File List
- File Members

# plugins/robots/foot-bot/simulator/footbot\_entity.cpp File Reference

`#include "footbot_entity.h"`  
`#include <argos3/core/utility/math/matrix/rotationmatrix3.h>`  
`#include <argos3/core/simulator/space/space.h>`  
`#include <argos3/core/simulator/entity/controllable_entity.h>`  
`#include <argos3/core/simulator/entity/embodied_entity.h>`  
`#include <argos3/plugins/simulator/entities/rab_equipped_entity.h>`  
`#include <argos3/plugins/simulator/entities/gripper_equipped_entity.h>`  
`#include <argos3/plugins/simulator/entities/ground_sensor_equipped_entity.h>`  
`#include <argos3/plugins/simulator/entities/led_equipped_entity.h>`  
`#include <argos3/plugins/simulator/entities/light_sensor_equipped_entity.h>`  
`#include <argos3/plugins/simulator/entities/omnidirectional_camera_equipped_entity.h>`  
`#include <argos3/plugins/simulator/entities/perspective_camera_equipped_entity.h>`  
`#include <argos3/plugins/simulator/entities/proximity_sensor_equipped_entity.h>`  
`#include <argos3/plugins/simulator/entities/wifi_equipped_entity.h>`  
`#include "footbot_distance_scanner_equipped_entity.h"`  
`#include "footbot_turret_entity.h"`  

Include dependency graph for footbot\_entity.cpp:

Go to the source code of this file.

|  |  |
| --- | --- |
| Namespaces | |
| namespace | argos |

|  |  |
| --- | --- |
|  | The namespace containing all the ARGoS related code. |

| Defines | |
| #define | UPDATE(COMPONENT)   if(COMPONENT->IsEnabled()) COMPONENT->Update(); |
| Functions | |
|  | argos::REGISTER\_ENTITY (CFootBotEntity,"foot-bot","Carlo Pinciroli [ilpincy@gmail.com]","1.0","The foot-bot robot, developed in the Swarmanoid project.","The foot-bot is a wheeled robot developed in the Swarmanoid Project. It is a\n""modular robot with a rich set of sensors and actuators. For more information,\n""refer to the dedicated web page\n""(http://www.swarmanoid.org/swarmanoid\_hardware.php).\n\n""REQUIRED XML CONFIGURATION\n\n"" <arena ...>\n"" ...\n"" <foot-bot id=\"fb0\">\n"" <body position=\"0.4,2.3,0.25\" orientation=\"45,0,0\" />\n"" <controller config=\"mycntrl\" />\n"" </foot-bot>\n"" ...\n"" </arena>\n\n""The 'id' attribute is necessary and must be unique among the entities. If two\n""entities share the same id, initialization aborts.\n""The 'body/position' attribute specifies the position of the bottom point of the\n""foot-bot in the arena. When the robot is untranslated and unrotated, the\n""bottom point is in the origin and it is defined as the middle point between\n""the two wheels on the XY plane and the lowest point of the robot on the Z\n""axis, that is the point where the wheels touch the floor. The attribute values\n""are in the X,Y,Z order.\n""The 'body/orientation' attribute specifies the orientation of the foot-bot. All\n""rotations are performed with respect to the bottom point. The order of the\n""angles is Z,Y,X, which means that the first number corresponds to the rotation\n""around the Z axis, the second around Y and the last around X. This reflects\n""the internal convention used in ARGoS, in which rotations are performed in\n""that order. Angles are expressed in degrees. When the robot is unrotated, it\n""is oriented along the X axis.\n""The 'controller/config' attribute is used to assign a controller to the\n""foot-bot. The value of the attribute must be set to the id of a previously\n""defined controller. Controllers are defined in the <controllers> XML subtree.\n\n""OPTIONAL XML CONFIGURATION\n\n""You can set the emission range of the range-and-bearing system. By default, a\n""message sent by a foot-bot can be received up to 3m. By using the 'rab\_range'\n""attribute, you can change it to, i.e., 4m as follows:\n\n"" <arena ...>\n"" ...\n"" <foot-bot id=\"fb0\" rab\_range=\"4\">\n"" <body position=\"0.4,2.3,0.25\" orientation=\"45,0,0\" />\n"" <controller config=\"mycntrl\" />\n"" </foot-bot>\n"" ...\n"" </arena>\n\n""You can also set the data sent at each time step through the range-and-bearing""system. By default, a message sent by a foot-bot is 10 bytes long. By using the""'rab\_data\_size' attribute, you can change it to, i.e., 20 bytes as follows:\n\n"" <arena ...>\n"" ...\n"" <foot-bot id=\"fb0\" rab\_data\_size=\"20\">\n"" <body position=\"0.4,2.3,0.25\" orientation=\"45,0,0\" />\n"" <controller config=\"mycntrl\" />\n"" </foot-bot>\n"" ...\n"" </arena>\n\n""You can also change the aperture of the omnidirectional camera. The aperture is\n""set to 70 degrees by default. The tip of the omnidirectional camera is placed on\n""top of the robot (h=0.289), and with an aperture of 70 degrees the range on the\n""ground is r=h\*tan(aperture)=0.289\*tan(70)=0.794m. To change the aperture to 80\n""degrees, use the 'omnidirectional\_camera\_aperture' as follows:\n\n"" <arena ...>\n"" ...\n"" <foot-bot id=\"fb0\" omnidirectional\_camera\_aperture=\"80\">\n"" <body position=\"0.4,2.3,0.25\" orientation=\"45,0,0\" />\n"" <controller config=\"mycntrl\" />\n"" </foot-bot>\n"" ...\n"" </arena>\n\n""Finally, you can change the parameters of the perspective camera. You can set\n""its direction, aperture, focal length, and range with the attributes\n""'perspective\_camera\_front', 'perspective\_camera\_aperture',\n""'perspective\_camera\_focal\_length', and 'perspective\_camera\_range', respectively.\n""The default values are: 'true' for front direction, 30 degrees for aperture,\n""0.035 for focal length, and 2 meters for range. When the direction is set to\n""'false', the camera looks up. This can be useful to see the eye-bot LEDs. Check\n""the following example:\n\n"" <arena ...>\n"" ...\n"" <foot-bot id=\"fb0\"\n"" perspective\_camera\_front=\"false\"\n"" perspective\_camera\_aperture=\"45\"\n"" perspective\_camera\_focal\_length=\"0.07\"\n"" perspective\_camera\_range=\"10\">\n"" <body position=\"0.4,2.3,0.25\" orientation=\"45,0,0\" />\n"" <controller config=\"mycntrl\" />\n"" </foot-bot>\n"" ...\n"" </arena>\n\n","Under development") |
|  | argos::REGISTER\_STANDARD\_SPACE\_OPERATIONS\_ON\_COMPOSABLE (CFootBotEntity) |

---

## Define Documentation

|  |  |  |  |  |  |
| --- | --- | --- | --- | --- | --- |
| #define UPDATE | ( | COMPONENT |  | ) | if(COMPONENT->IsEnabled()) COMPONENT->Update(); |

Definition at line 449 of file footbot\_entity.cpp.

---

Generated on 10 Jul 2018 for ARGoS by 
 1.6.1
